# Precision DNA Mixture Interpretation with Single-Cell Profiling

**DOI:** 10.3390/genes12111649

**Published:** 2021-10-20

**Authors:** Jianye Ge, Jonathan L. King, Amy Smuts, Bruce Budowle

**Affiliations:** 1 Center for Human Identification, University of North Texas Health Science Center, Fort Worth, TX 76107, USA; Jonathan.King@unthsc.edu (J.L.K.); Amy.Smuts@unthsc.edu (A.S.); Bruce.Budowle@unthsc.edu (B.B.); 2 Department of Microbiology, Immunology and Genetics, University of North Texas Health Science Center, Fort Worth, TX 76107, USA

**Keywords:** DNA forensics, DNA mixture, mixture interpretation, single-cell, clustering algorithm, number of contributors, consensus profile

## Abstract

Wet-lab based studies have exploited emerging single-cell technologies to address the challenges of interpreting forensic mixture evidence. However, little effort has been dedicated to developing a systematic approach to interpreting the single-cell profiles derived from the mixtures. This study is the first attempt to develop a comprehensive interpretation workflow in which single-cell profiles from mixtures are interpreted individually and holistically. In this approach, the genotypes from each cell are assessed, the number of contributors (NOC) of the single-cell profiles is estimated, followed by developing a consensus profile of each contributor, and finally the consensus profile(s) can be used for a DNA database search or comparing with known profiles to determine their potential sources. The potential of this single-cell interpretation workflow was assessed by simulation with various mixture scenarios and empirical allele drop-out and drop-in rates, the accuracies of estimating the NOC, the accuracies of recovering the true alleles by consensus, and the capabilities of deconvolving mixtures with related contributors. The results support that the single-cell based mixture interpretation can provide a precision that cannot beachieved with current standard CE-STR analyses. A new paradigm for mixture interpretation is available to enhance the interpretation of forensic genetic casework.

## 1. Introduction

Interpreting DNA mixtures is one of the most challenging problems in forensics genetics. The current standard workflow and mixture interpretation processes are used to extract DNA from a crime scene sample (e.g., swabs), quantify the extracted DNA (although in some protocols this step can be skipped), amplify targeted Short Tandem Repeat (STR) regions, detect DNA fragments through Capillary Electrophoresis (CE), call alleles with accompanying software, and interpret or deconvolve the contributors’ DNA profiles as best as is possible from the collection of allelic peaks in an electropherogram primarily by a DNA analyst(s) based on training and experience with or without the assistance of probabilistic genotyping software programs (such as STRmix [1], LRmix [2], TrueAllele [3]). With this generalized CE-STR analysis process—although the DNA may still be contained in individual cells when collected—during extraction the cells, and thus the DNA, are pooled. If there is more than one contributor to the sample, a mixture is obtained. Subsequent to generating the mixture profile, an analyst(s) attempts to decipher the information to determine, for example, the number of contributors (NOC) in a mixture, the genotypes of individual contributors, if a particular individual is or is not a contributor of a mixture, and so forth. Given the available information generated through this standard process, the mixture profile is usually interpreted by indirect methods, such as inferring the NOC by counting the observed allelic peaks or evaluating the weight of the evidence assuming a person as being a contributor vs. an unknown person being a contributor by a likelihood ratio (LR) approach. The LR compares the likelihoods of observing the same evidence given two or more competing hypotheses (e.g., the mixture is composed of the victim and the suspect vs. the mixture is composed of the victim and a random person in a population). At times, the deconvolution of the contributing genotypes can be very challenging because of overlapping alleles, allele drop-out (ADO), allele drop-in (ADI), and uncertainty in the NOC. All forensic genetic methods to date, and likely for the foreseeable future, suffer from these phenomena. For example, it can be formidable with the current standard analysis to determine the NOC of a three-person mixture formed by two parents and their child, since both alleles of the child are shared with the parents. In addition, it is difficult to determine if a peak in a stutter position of a major contributor allele is composed of an allele from a minor contributor and stutter or solely a stutter product. Thus, some details of a mixture may not be able to be ascertained with a high degree of certainty.

To reduce the limitations of current standard mixture analyses, alternate methods have been proposed, such as enzymatic digestion differential extraction [4,5], reducing the allele overlap by using sequence-based alleles instead of traditional length-based alleles [6], employing probabilistic genotyping software [1,2,3], and so forth. While these alternate methods have added some value for mixture interpretation, the recent single-cell detection and analysis technologies may also reduce the challenges of DNA mixture interpretation. In theory, no mixture is generated as each cell is independently analyzed and thus the data from a single cell are derived from a single contributor. The DNA quantity of the contributors of a mixture can be more precisely determined by counting the isolated cells and cell types; the mixture ratio can be easily determined even though ratios may not be necessary anymore; and the alleles of each single cell can be called independently. The interpretation of these results is fundamentally different from the current interpretation approaches but should be much simpler. In other words, the computational intensive probabilistic genotyping methods, which are notably superior to previous manual methods, “guess”, and often quite well, the possible genotypes of the contributors [1,2,3]; instead, single-cell profiles can be directly compared with the reference/known profiles to support whether or not an individual is a contributor of a mixture, if a good quality profile is obtained. However, due to technology limitations, a single-cell DNA profile usually contains ADO and/or ADI; in such scenarios, the consensus profiles from clustered cells can be used in single source profile comparisons or database searches. The consensus methods have been successfully used in single cell RNA studies [7,8] and forensic applications [9,10]. Therefore, this approach can reduce uncertainty regarding the profiles contributing to a mixture and thus provide stronger support for investigative leads and the judicial processes.

In a single-cell analysis workflow, cells are individually isolated from forensic samples. There are several methods developed that are capable of isolating single cells, such as manual micromanipulation [11,12,13,14,15,16,17], Laser Capture Microdissection (LCM) [18,19], Magnetic Activated Cell Sorting (MACS) flow cytometry [20], Fluorescent Activated Cell Sorting (FACS) flow cytometry [21,22,23,24,25], and a dielectrophoresis system (e.g., DEPArray) [26,27,28]. After isolation, the single cells can be individually amplified for the targeted regions (e.g., STRs) or subjected to Whole Genome Amplification (WGA) before amplification to enrich the DNA targets [17,29,30,31,32,33].

Li et al. [34] used the Identifiler kit (Thermo Fisher Scientific, South San Francisco, CA, USA) to amplify 15 autosomal STRs from 20 single sperm cells from one donor, and Han et al. [19] performed a similar study on 37 single sperm cells (from three donors) typing ten autosomal STRs with amplicon sizes <300 bp. In both studies, the consensus genotypes were consistent with the known donor genotypes. In Li et al. [34], the ADO rate was 25% and the ADI rate was 1.3%. In Han et al. [19] the ADO rate was lower (15%), possibly because the overall amplicon sizes were shorter, and the ADI rate was similar (1.4%). In these studies, the ADIs did not all reside at stutter positions of the true alleles. Some ADIs may have been due to DNA fragments from the other lysed cells which were co-amplified with a single cell and/or some ADIs might be due to genotype errors or low-level contamination. Anslinger et al. [27] collected 11 white blood cells with the DEPArray and genotyped 16 autosomal STRs using the PowerPlex ESXfast kit (Promega Corp., Madison, WI, USA). They found that 82% of alleles were obtained (i.e., 18% ADO rate), only one ADI was observed at a stutter position (i.e., 0.3% ADI rate), and the full profile was recovered by consensus. Even with ADO and ADI, full profiles of the donors were obtained by consensus of the single-cell profiles.

A recent study by Chen et al. [33] employed a WGA kit (Qiagen REPLI-g single cell kit, Hilden, Germany) and subsequently typed the products with both the GlobalFiler kit (Thermo Fisher Scientific, South San Francisco, CA, USA) on the CE platform and the ForenSeq DNA Signature Prep kit (Verogen, San Diego, CA, USA), on a Massive Parallel Sequencing (MPS) platform (i.e., MiSeq FGx, Verogen, San Diego, CA, USA). For the three single cells analyzed with the GlobalFiler kit only four STR alleles dropped out (i.e., 3.2% ADO rate), and no ADI was observed. For the three single cells amplified with the ForenSeq kit, all autosomal STR alleles were successfully recovered without any ADI. The better performance of the ForenSeq kit, compared with that of the GlobalFiler kit, may be due to the overall shorter amplicon sizes of the STR alleles and/or a different way noise is presented with MPS data. Deleye et al. [31] recently compared four commercial WGA kits, Ampli1 (Menarini Silicon Biosystems, Castel Maggiore, BO, Italy), DOPlify (PerkinElmer, Waltham, MA, USA), PicoPLEX (Takara Bio, Kusatsu, Japan), and REPLI-g (Hilden, Germany) with single B-lymphoblastoid cells and found that the REPLI-g had the lowest ADO rate for STR profiling (i.e., 8.33%), while the other kits had ADO rates varying from 35.71%–60.71%. According to Chen et al. [35], the major commercial WGA kits yield various genome coverages (41%–92%) and ADO rates (25%–65%). Thus, it is reasonable to assume that substantial ADO and ADI would be observed with single-cell WGA per cell if a larger number of single cells are sampled.

All these studies demonstrate that single-cell technologies can recover the STR alleles of single cells with a high success rate. However, to date, no study has provided a systematic approach to interpret the STR data from single-cells derived from mixtures. This study is the first attempt to develop and assess performance of interpretation methods for characterizing multiple donors from single-cell profiles of mixtures. The study herein evaluates these interpretation methods by a simulation approach with various mixture scenarios and reasonable ADO and ADI rates, and provides data in terms of the number of cells, NOC, and ADO and ADI rates. Through this study, the potential of the single-cell technology workflow was evaluated for the probabilities of not detecting a minor contributor during cell sampling, the accuracies of estimating the NOC with single cells and sampling, the accuracies of recovering the true alleles by single-cell profile consensus, and the capabilities of single-cell approach for deconvolving mixtures with related contributors. The results convey the high precision that a single cell workflow could achieve for mixture interpretation.

## 2. Methods

### 2.1. Simulation Methods

The autosomal STR genotypes of the single diploid cells (e.g., epithelial cells) were simulated using the method described in [36,37] for mixtures with unrelated and related contributors. In the simulation, the genotype of each marker of an individual was randomly simulated based on the allele frequencies and the population substructure correction (i.e., F_st_) following Balding et al. [38]; the markers were assumed to be independent; the genotypes of related individuals were simulated assuming the parents transmit with equal probability a single allele at each locus to their children; the Two-Phase mutation model [39] was employed in the parent–child allelic transmissions; the mutation rates in the AABB report [40] were used. First, the genotype profile of each contributor in a mixture was simulated for the 21 autosomal STR loci in the GlobalFiler PCR amplification kit [41] using Caucasian population allele frequency data [42] and a F_st_ of 0.01. Second, based on the simulated genotype profiles of the contributors, ADO and ADI were incorporated to simulate the profiles of every single cell with a given number of cells of the contributors (e.g., 20 cells of contributor A and 20 cells of contributor B). Third, a mixture was formed by pooling the single-cell profiles of the contributors (e.g., a mixture with 40 cells in total from contributors A and B). Multiple mixtures with various mixture ratios were simulated, including mixtures with either unrelated (UR) or related contributors, namely, parent-child (PC), full-sibling (FS), and standard family trio (Table 1). Supplementary Table S1 gives an example of a simulated mixture with two unrelated contributors (20 cells each) in the ARFF format defined by WEKA [43], in which one attribute represents one allele of a locus (i.e., two attributes for a diploid genotype). The smaller allele was always assigned as the first allele for a heterozygous genotype, and alleles were listed in a defined attribute order.

The ADO model described in Balding et al. [44] was used in the simulation, in which *D* represents the probability of drop-out of one allele at a heterozygous locus, and *D_2_* represents the probability of drop-out at a homozygous locus, with *D_2_* ≈ ½*D^2^*. The rare scenarios that a locus of a diploid cell or a haploid cell presents more than two alleles or more than one allele, respectively, were ignored in this study (i.e., only up to two alleles or one allele with strongest signal(s) for diploid and haploid cells, respectively, would be called), allele size dependent degradation was ignored, and somatic mutations (i.e., the genotypes of the diploid cells from the same contributor may be different) were also ignored. The ADI was incorporated as a random genotyping error (with an ADI rate of *e*); namely, one allele may be randomly called as any other allele at this locus in the population data with equal chance. For example, changes to genotypes 10,10 → 10,15 or 10,11 → 10,15 for a homozygous and heterozygous locus, respectively, for diploid cells were produced to approximate ADI. Thus, a heterozygous locus with ADI has to have one ADO involved. However, the ADO and ADI events in general were assumed to be independent for the purposes of this study. When the ADO and ADI rates are 0, the genotype profiles of single diploid cells from the same contributor should be identical.

For the profiles of the single haploid cells the interpretation is slightly different because only one allele out of two at each autosomal locus is randomly distributed in the haploid cells. Therefore, the interpretation of haploid single-cell mixture profiles may be more complicated than that of diploid cells, even without ADO or ADI. In the haploid profile simulations, the alleles were generated by randomly selecting one allele of the two simulated diploid alleles at each locus and assuming independence among the loci. The same population data, parameters, ADO model, and ADI model as in the diploid simulations were used in simulating the haploid single-cell profiles.

### 2.2. Clustering Single Cells

To estimate the NOC of a mixture and allele consensus of each contributor, the single cells need to be grouped into clusters (or contributors). There is a large number of clustering algorithms that may serve this purpose. This study tested two classic clustering algorithms, Expectation–Maximization (EM) [45,46] and K-Means [47]. The EM algorithm assigns a probability distribution for the latent variables to each instance in the dataset (i.e., each single cell), which indicates the probability of it belonging to each of the clusters (i.e., each contributor in a mixture), computes parameters to optimize the likelihood of the model, and then repeats these two steps until convergence [43,45]. The EM algorithm implemented in WEKA uses multivariate Gaussians with diagonal covariance matrices as mixture components [46]. The EM algorithm does not explicitly require a distance measure between the instances. The K-Means algorithm randomly chooses an initial centroid for each cluster, each instance is assigned to its nearest centroid to form clusters, the centroids are updated with the instances in the same clusters, and these two steps are repeated until convergence [47]. The K-Means algorithm requires a distance measure between instances, which is Euclidean distance by default. The EM and SimpleKMeans algorithm implementations with default setting in the WEKA software package [43] were used in this study.

The clustering algorithms usually require a predefined number of clusters. To determine the best number of clusters, the Silhouettes method [48] implemented in ClusterMaker [49] was used. For a given number of clusters, the Silhouettes coefficient measures how similar an instance is to its own cluster compared to other clusters. The maximum value of the Silhouettes coefficient indicates the most likely number of clusters for a given range of the number of clusters [50]. For the mixtures in Table 1, the Silhouettes coefficients of two to six clusters were calculated and compared, and the number of clusters with the highest Silhouettes coefficient was determined as the NOC.

### 2.3. Single-Cell Profile Visualization

Multidimensional Scaling (MDS) was used to visualize the clustering of single-cell profiles. The three-dimensional (3D) MDS coordinates of every single-cell profile of a mixture were calculated using MDSJ [51], in which the distance between two profiles was measured as the number of mismatched alleles normalized by the total number of alleles. Then, the 3D coordinates were visualized by JTableSaw [52].

### 2.4. Consensus Method

For the single-cell profiles clustered into the same cluster, the genotype profiles of these single cells were determined by consensus and then compared with the true genotypes to assess the accuracies of recovering the true alleles by the clustering approach. For both diploid and haploid cells, the consensus of each allele was determined independently (i.e., alleles at the same position defined in the order as described in Section 2.1 and Supplementary Table S1). The consensus allele at a position (e.g., the first allele of the vWA locus in a diploid cell, or the only allele of the vWA locus in a haploid cell) in a cluster was defined as the most frequent allele at this position among the single-cell profiles in this cluster. The missing data due to ADO were ignored during consensus.

## 3. Results

### 3.1. The Probability of Not Detecting a Contributor during Sampling

The current standard CE-STR mixture analysis process may not be able to provide a precise estimate of the DNA input quantities of the contributors, nor the probability that a minor contributor is not observed in a mixture. The limitations are due in part to only the contributors with sufficient input quantities showing allele peaks above a detection threshold, and allele sharing may mask a minor contributor; these phenomena become more exacerbating as the number of contributors increases. Thus, a minor contributor may not be detected through this process. With the single-cell analysis, however, the quantity of a sample can be directly determined by counting the cells in a sample, although sampling error can occur during cell isolation. Therefore, the probability of not detecting a minor contributor can be estimated with the single-cell profiling.

Assuming a large number of cells are available in a forensic sample (i.e., the sampling process follows a binomial distribution) and each cell has an equal chance to be sampled, the probability that none of the cells from a contributor with a mixture proportion of *p* is sampled in the total sampled *n* cells is *(1-p)^n^*. Table 2 estimates these probabilities for various mixture proportions of a contributor(s) and different total numbers of sampled cells, either diploid or haploid cells, with 6.6 pg DNA for a diploid cell and 3.3 pg DNA for a haploid cell [53]. The mixtures containing both diploid and haploid cells (e.g., a mixture of epithelial cells and sperm cells) were not considered (but can be readily determined if desired); these cellular mixtures can be separated either by counting the number of alleles across the loci or by technologies (e.g., DEPArray) to select cells from different tissues.

In general, as more cells are sampled the chance of not detecting a contributor decreases. For example, if 80 diploid cells are sampled (~528 pg DNA in total, close to the recommended DNA input of some commercial CE-STR kits, that is, 500 pg), there is a 44.75% chance that none of the cells from a 1% minor contributor is sampled; however, for a 5% minor contributor there is only a 1.65% chance of not detecting the minor contributor. For a low quantity DNA sample with only 15 cells (~99 pg), there is a 46.33% chance that a 5% minor contributor would not be detected. To obtain a high confidence of detecting a minor contributor with 1% or lower proportion, ≥500 single cells may need to be sampled.

### 3.2. The Accuracies of NOC Estimation

#### 3.2.1. NOC Estimation by Clustering

The two-person and three-person mixtures were simulated with various numbers of cells, various mixture ratios, unrelated or related contributors, and both diploid and haploid cells (Table 1). In the simulation, the ADO and ADI rates were set as 0.2 and 0.01, respectively, which were similar to the estimations of several studies [19,27,34] and reflected the current performance of single-cell studies. For each mixture scenario, 10,000 cases were simulated, and the NOC for each case was estimated by the EM and K-Means clustering algorithms, together with the Silhouettes method. Table 1 shows the accuracies of estimating the NOC for the simulated mixture scenarios with the EM algorithm, which provides higher accuracies compared with the K-Means algorithm. The NOC accuracies estimated by K-Means are listed in Supplementary Table S2.

For diploid cells, in general, higher accuracies were achieved with more cells and/or more balanced mixture ratios. If contributors were unrelated, almost all scenarios had >98% accuracy, except for three-person mixtures with a very few number of cells sampled and severely imbalanced mixture ratios (e.g., 2,6,12 and 1,3,6). Almost 100% accuracies were obtained if the contributors were unrelated, and there were more than 20 cells in total sampled. For PC and FS pairs, most of the estimated accuracies were higher than 98%, although, as expected, lower than those of UR pairs. Estimating the NOC for a mixture composed of a family trio can be particularly challenging with CE-STR analyses as well as with MPS; however, the accuracies with single cell analyses and the EM algorithm together with Silhouettes method were mostly >80%, except for scenarios with an extremely low number of sampled cells (e.g., ten cells) or highly imbalanced mixture ratios (e.g., 1:3:6).

The NOC accuracies with haploid cells were lower than those of diploid cells, because the same contributor may have many different haploid profiles due to meiosis, and the ADO and ADI would add more variants making the clustering of haploid cells more challenging. However, even with these limitations, high accuracies were achieved when the mixture ratios were relatively balanced and the number of sampled cells was high (e.g., 98.17% accuracy with a (53,27) Parent-Child mixture). Even for highly imbalanced mixtures (for example, 72,8), NOC accuracies were 95.56% and 85.95% for UR and PC contributors, respectively, which were still reasonably high.

#### 3.2.2. Impact of ADO and ADI on NOC Estimation by Clustering

**Figure 1:**
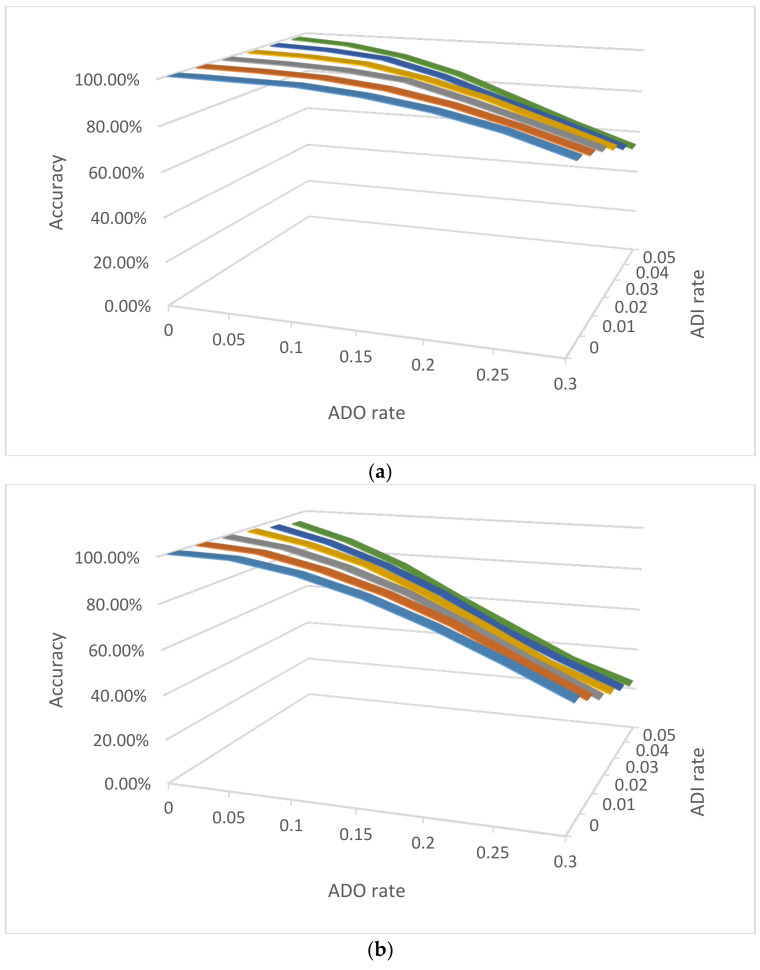

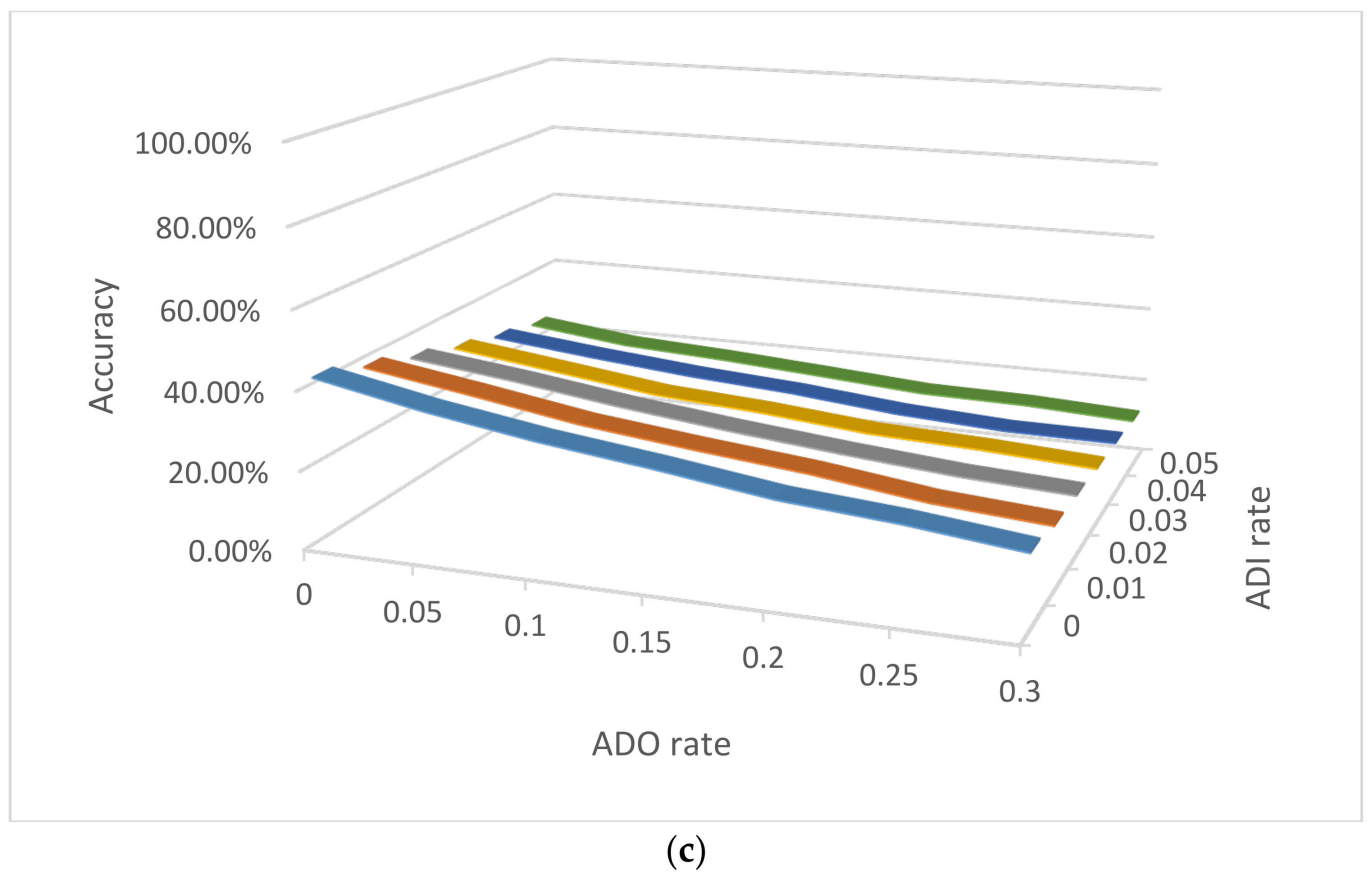
The accuracies of NOC estimation for 3-person mixtures with 2, 6, and 12 single cells with various ADO and ADI rates in three scenarios: (**a**) Diploid cells with three unrelated contributors, (**b**) Diploid cells with three family trio contributors (father, mother, and child), and (**c**) Haploid cells with three unrelated contributors. *D* = 0.2; *e* = 0.01; 10,000 simulations for each mixture scenario.

The accuracies of estimating the NOC can be impacted by the ADO and ADI rates. To investigate the impact of ADO and ADI, the same simulations as above were conducted for three mixture examples (Figure 1) with the same number of contributor cells (two, six, and 12 cells) for a three-person mixture, with different relationships among the contributors, different cell types, and various ADO and ADI rates.

**Figure 2:**
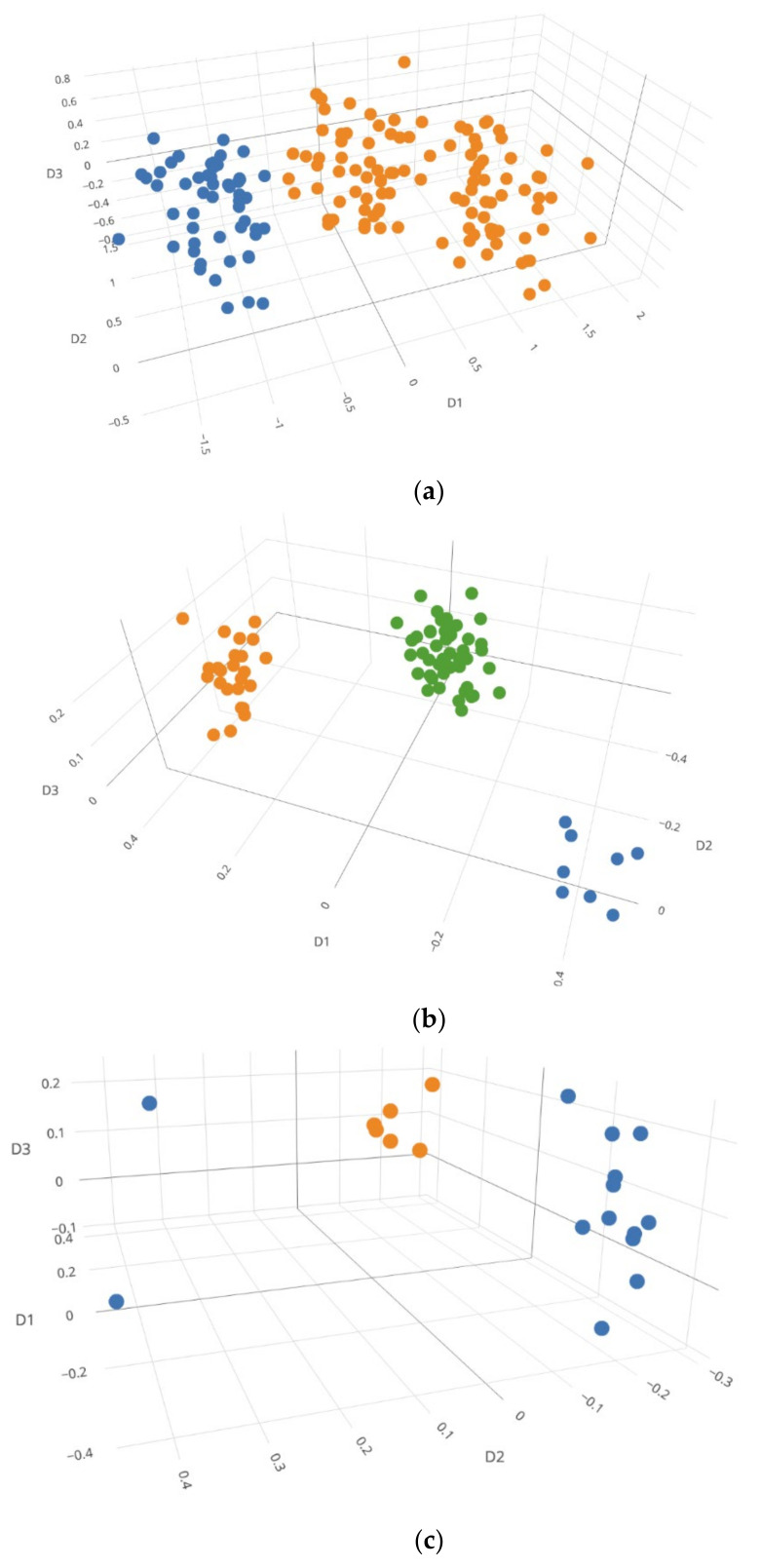

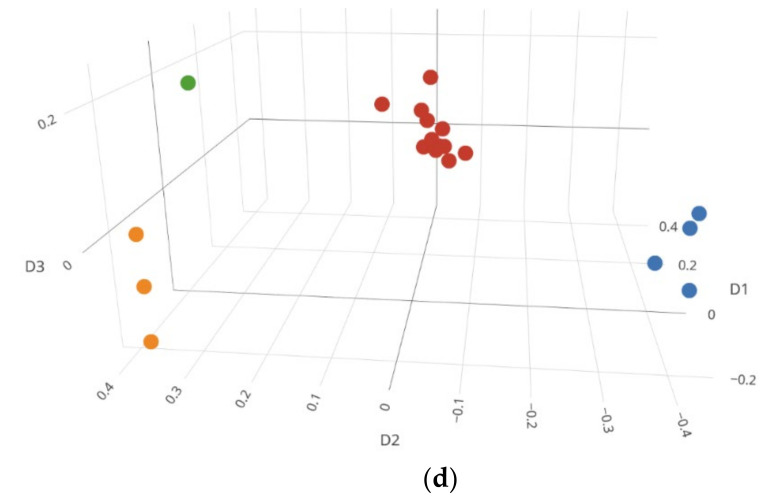
Mixture visualization examples with 3D MDS plots: (**a**) A family trio diploid cell mixture (53, 53, 53) incorrectly clustered with the EM algorithm and Silhouettes method as two contributors. (**b**) A family trio diploid cell mixture (8,24,48) correctly clustered with the EM algorithm and Silhouettes method as three contributors. (**c**) A 3-person haploid cell mixture (2,6,12) incorrectly clustered with the EM algorithm and Silhouettes method as two contributors. (**d**) A 3-person diploid cell mixture (4, 4, 12) incorrectly clustered with the EM algorithm and Silhouettes method as four contributors. Each color represents each cluster. Rotatable plots of these figures can be found in Supplementary Figure S1.

For diploid unrelated cells (Figure 1a), the accuracies with lower ADO rates are higher and decrease with increasing ADO rates (e.g., 99.16% with *D* = 0.05 and *e* = 0.01, compared with 89.88% with *D* = 0.2 and *e* = 0.01). The ADI had less impact with the NOC accuracies due to the relatively low ADI rates (i.e., at the level of 0.01) compared with relatively high ADO rates (i.e., at the level of 0.1). The accuracies dropped more so with family trio mixtures (Figure 1b), likely because the related contributors share at least half of their alleles (assuming no mutation), and ADO and ADI could contribute to some cells from a different contributor sharing more alleles than expected, and eventually blur the boundaries between contributors (see Figure 2a as an example). The haploid mixtures, as expected, had a lower estimation of NOC accuracies. Even with no ADO or ADI, the accuracy was low (i.e., 41.38%), which is mostly due to the varied haploid profiles from each cell of the same contributor, a small number of sampled cells, and poor performance of the EM algorithm for imbalanced mixtures. Sampling more cells, as well as algorithms better designed specifically for imbalanced single-cell genotype data, should improve the accuracies.

#### 3.2.3. NOC Estimation by Visualization

Among these tested mixtures, the accuracies dropped substantially when the mixture ratios were extremely imbalanced (i.e., 9:1 and 19:1), which is mostly because the classic clustering algorithms do not perform well with imbalanced data (i.e., large sample size differences between or among clusters) [54]. The related contributors (i.e., the data points are closer to each other), haploid data (i.e., genotype uncertainty), and small numbers of cells (i.e., clustering with sparse data) can further reduce the accuracies. In such scenarios, particularly when the number of sampled cells is small, visualization of the distance between and among single-cell profiles may facilitate human judgement on the NOC. Visualization has been shown to be an effective way to estimate the NOC and differentiate cell types in single-cell applications [55,56].

Figure 2 displays several examples of 3D MDS plots. In Figure 2a, the EM algorithm and Silhouettes method together incorrectly determined a family trio mixture as a two-person mixture, because many cells of two specific contributors (i.e., one of the parents and the child) were very close to each other and the algorithm considered them as one contributor. However, with visualization, the NOC most likely could be determined as three. Figure 2b is an example with a correct NOC estimation, in which all contributors were clearly separated. In Figure 2c, two contributors (2 and 12) were incorrectly clustered into a single contributor, but with the MDS plot they appear separated. In Figure 2d, a three-person mixture was incorrectly determined as a four-person mixture. In one of the minor contributors, one single-cell profile was distant from the other three single-cells, which led to an incorrect NOC estimation. Even with MDS plot, it was challenging to determine the NOC, as both three and four clusters appeared possible.

#### 3.2.4. NOC Estimation by Identity-by-State (IBS) Distance Measure

**Figure 3:**
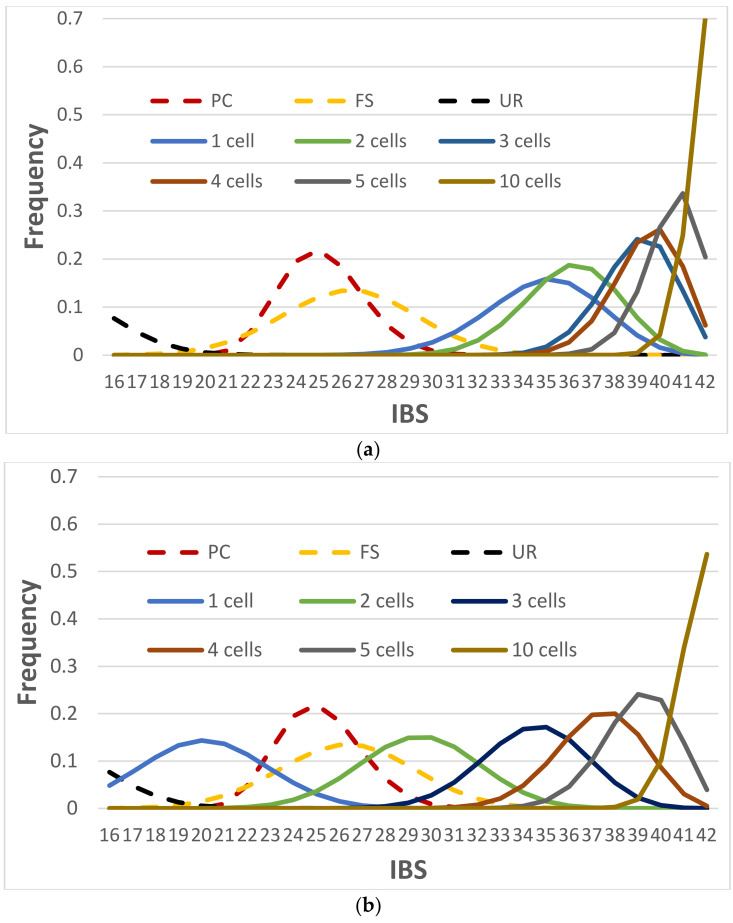
IBS distributions of unrelated and related pairs (UR, PC, and FS, in dotted lines) and pairs of the true profile versus the consensus profile with a given number of cells (in solid lines) based on (**a**) diploid cells and (**b**) haploid cells. *D* = 0.2; *e* = 0.01; 1,000,000 simulations for each distribution. The IBS of “*n* cell(s)” is the IBS between true genotypes and the consensus genotypes from *n* cell(s). These distributions are based on 21 GlobalFiler markers and thus the maximum IBS is 42. The consensus profile might contain missing alleles, and these missing alleles were excluded in counting IBS.

Another way to estimate the NOC is to measure the distances between single-cell profiles using predefined IBS thresholds. Figure 3 shows the IBS distributions of unrelated and related pairs of individuals (UR, PC, and FS) and pairs of the known alleles of a contributor versus the consensus alleles with a given number of sampled cells, assuming the clustered profiles were from the same contributor. Apparently, there was no overlap between the IBS distribution of the unrelated and one cell with diploid cells, which means two unrelated diploid cells can be clearly separated simply by a reasonable IBS threshold (e.g., IBS ≤ 24 for the scenario of *D* = 0.2 and *e* = 0.01). This simple approach can readily determine if a group of single cells is from one contributor or multiple unrelated contributors (i.e., single source or mixture), as well as if a cell profile and a known profile from an unrelated individual may be from the same source. Thus, the NOC can be precisely determined by measuring the IBS distance between every pair of diploid cell profiles and a predefined maximum IBS distance for cells within the same cluster (e.g., IBS ≤ 24), assuming all contributors are unrelated. However, this approach may not work well for haploid cell profiles, because the consensus diploid profile relying on a single haploid cell would be homozygous across the loci. There would be a good proportion of the IBS distributions of UR and 1 haploid cell profile that would overlap. For example, 9.6% of unrelated pairs had IBS ≥ 16, and 9.7% of the consensus profiles from one haploid cell had IBS ≤ 17 (Figure 3b).

If the contributors are related, the IBS measure may not reach 100% accuracy to differentiate two unrelated cells from two related single cells (i.e., there were overlaps between the “one cell” distribution and the FS or PC distributions (Figure 3)). However, if multiple cells could be associated by either clustering, measured distances, or visualization, the consensus profile could differentiate a profile from his/her parent or full-siblings simply by IBS counting (e.g., a false rate less than 0.05% with a consensus profile from five cells for both PC and FS, and almost no error with ten cells (Figure 3)), which was similar to haploid cells (Figure 3b).

### 3.3. Accuracies of Consensus

#### 3.3.1. IBS Distributions with Consensus

Once the NOC is estimated for a mixture, the consensus profile of each contributor can be generated by common alleles of the clustered single cells. As shown in Figure 3a, one single diploid cell as a single contributor mostly shared at least 28 alleles (with an average of 35 alleles) with the true profile, and the mismatches were mostly due to the high ADO rate (i.e., 0.2). Thus, there is sufficient precision to differentiate one single-cell profile from a random person profile (~0.03% average error rate with a threshold of IBS ≤ 24; the average error rate was the average of the false positive rate of determining a consensus profile and an unrelated profile as from the same source, and the false negative rate of determining the two profiles as being from different sources).With more cell profiles clustering in the same contributor, more shared alleles are found between the consensus profile and the true profile.

The consensus of haploid cell profiles would be more challenging, but would have more success than the current standard process. It is unlikely to determine the source of a single haploid cell profile (Figure 3b). However, the consensus profile of two haploid cells in a cluster, if they truly belong to the contributor, could be differentiated from a random person (<0.13% average error rate with a threshold of IBS ≤ 21), even though, on average, ~30% of the consensus alleles might not be correct (i.e., different from the true alleles or missing). For a contributor with ≥1 haploid cells, the consensus profile most likely would have ≤3 inconsistent alleles compared with the true profile. In general, these results were consistent with the simulation study by Lucy et al. [57], although ADO and ADI were not included in their study.

In addition, the results in Figure 3 indicated that the mixture ratio estimation of the sperm samples with the current CE-STR analysis might not be accurate for the extremely low quantity DNA samples, even if there is no ADO, ADI, or degradation. Each sperm cell has only one allele at a locus, and a contributor with a few sperm cells may have highly imbalanced allele sampling. For example, three out of four sampled sperm cells may share one particular allele of a heterozygote and the other sperm could have a different allele, which could make this contributor appear as a 3:1 mixture with two homozygotes. The mixture ratio at different loci may randomly vary when the number of sampled haploid cells is small, which likely could lead to an incorrect interpretation or more alternate genotypes to consider with the CE-STR mixture analysis.

#### 3.3.2. A Suspect or His/Her Close Relatives as Contributors

In certain cases, it may be necessary to determine if a suspect or one of the suspect’s close relatives is a potential contributor to a mixture. Based on the IBS distributions in Figure 3a, with only a few diploid cell profiles, an IBS threshold of ≤34 can precisely differentiate the related individuals if a consensus profile is from a known profile or a full-sibling of this known profile. With only three diploid cell profiles, 0.68% of the consensus profiles would have an IBS ≤ 34 (i.e., false negatives), 0.18% of the FS pairs would have IBS ≥ 35 (i.e., false positives or more appropriately adventitious associations). Sampling more cells can reduce the false rates. For haploid mixtures, if the same threshold is used (i.e., IBS ≤ 34), five haploid cell profiles would be needed to achieve high accuracy (i.e., 0.64% false negative rate). Thus, it only requires a consensus accuracy as low as 83.3% (35/42) to precisely differentiate if a consensus profile is from a known individual (e.g., suspect) or this individual’s first-degree relative(s).

The average accuracies of consensus for all diploid mixtures and most haploid mixtures with balanced contributors were higher than 83.3% (Table 3). Therefore, except for minor contributors with ≤2 diploid cells or ≤4 haploid cells sampled, the consensus profiles of each contributor in the mixtures should be able to determine the source of the profile with very high accuracies (Table 3). Again, more cells sampled would increase these accuracies.

#### 3.3.3. Accuracies of Consensus by Clustering

The distributions in Figure 3 assumed the cells clustered into contributors were truly the cells from this contributor. In fact, cells may be clustered to an incorrect contributor. However, the incorrectly clustered cells should be overwhelmed (assuming a sufficient number) by the cells correctly belonging to a contributor and the incorrect alleles should be filtered out by consensus, if a good clustering algorithm is used and there are enough cells clustered to this contributor.

To test this hypothesis, the consensus accuracies of clustering results were estimated using the same simulations conducted as in Table 1, and the average accuracies of consensus alleles were calculated for each simulated mixture. For example, a mixture with two contributors may have consensus accuracies of 0.8 and 0.9, respectively, and the average accuracy would be 0.85. For mixtures with an imbalanced mixture ratio (e.g., 1:3:6), the minor contributor(s) usually had lower consensus accuracies, mostly due to the small numbers of sampled cells. Table 3 shows the average of 10,000 simulations for the average accuracies of the consensus profile. Only the cases with the correct NOC estimation were included in these calculations.

As expected, given the correct NOC estimation, mixtures with a relatively high number of cells have very high consensus accuracies. Full profiles can be precisely obtained by consensus of the cells in the same clusters, if there are enough sampled diploid cells for a mixture with relatively balanced contributor ratios. The mixtures with relatively low consensus accuracies were mainly due to the low accuracies of the minor contributors with really low numbers of sampled cells. For example, the mixtures with (152,8) had an average accuracy of 98.01%, but the average consensus accuracy of the major contributor (i.e., 152) was 100%, but the minor contributor (i.e., eight) had a lower accuracy (i.e., ~96%).

Even with imbalanced contributors, all mixtures with at least five diploid cell profiles for the minor contributors achieved >96% average consensus accuracy, mostly greater than 97.6% (i.e., one allele mismatch or missing, 41/42). Particularly, the individual profiles of the family trio mixtures could be recovered even with only 40 sampled cells and imbalanced contributors, if one allele mismatch or missing was allowed. These observations were consistent with those in Figure 3, which indicates that consensus was able to filter out most incorrect alleles, if not all, caused by either ADO and ADI or incorrect clustering. These consensus profiles with just a few mismatched or missing alleles should be precise enough to generate reasonable partial matches (or hits) in a forensic DNA database (e.g., CODIS) search and support the potential identities of the contributors.

The consensus accuracies of the haploid cell mixtures, in line with expectations, were lower than those of diploid cell mixtures. However, the accuracies were still high for mixtures with enough sampled cells (e.g., ≥20 cells) and relatively balanced contributors, even with related contributors (e.g., 94.51% for FS mixtures of 13:7). Similar to the NOC estimation with haploid cell profiles, the accuracy dropped substantially for extremely imbalanced contributors. This observation is most likely because the EM algorithm does not perform well for imbalanced data. When the number of cells of a cluster is few (e.g., ≤4), together with ADI and relatively high ADO, 1 or 2 incorrectly clustered cells may dramatically change the consensus alleles. Better clustering algorithms specifically designed for imbalanced data may be able to improve the consensus accuracies.

Moreover, the relationships between the contributors appear to have little impact on the consensus accuracies when the number of cell profiles of each contributor is high (e.g., ≥16) or the contributors are relatively balanced; the consensus accuracies of unrelated and related contributors of the mixtures were almost identical for diploid cells (Table 3). In other words, the relationships between the contributors could impact the NOC estimation, but once NOC was correctly estimated, the consensus within each cluster solely relied on the profiles in the same cluster. However, when the number of cells from a contributor is small, the consensus accuracies vary more so, since a few incorrectly clustered cells may change the consensus alleles.

**Figure 4:**
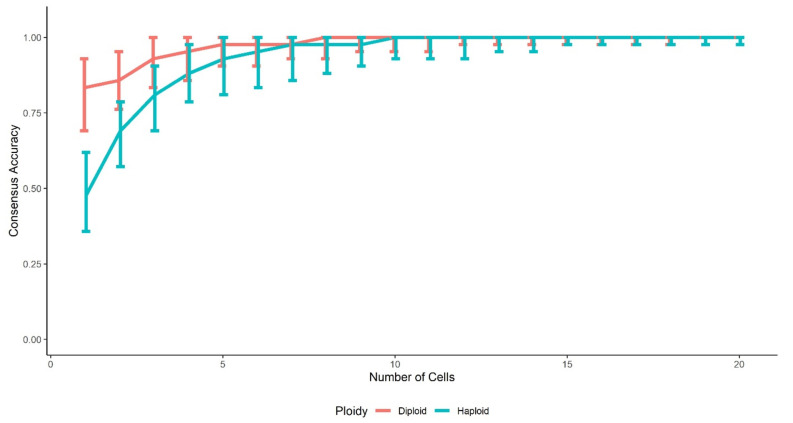
Consensus accuracy as a function of the number of cells in the cluster, with 1:1 ratio mixtures. *D* = 0.2; *e* = 0.01; 10,000 simulations for each mixture. The bars represent the 95% confidence intervals.

In addition, the two-person mixtures with 1:1 ratio were used as examples to investigate the consensus accuracy as a function of the number of cells in the cluster (Figure 4), for both diploid and haploid cells. The accuracies increase fastly with more cells added into the same clusters when the number of cells in a cluster is relatively small. With these balanced mixtures, a cluster with ≥5 diploid cells or ≥7 haploid cells had ≥97.6% accuracy (i.e., one allele mismatched or missing), which was consistent with the results in Table 3. For the extremely imbalanced mixtures, the consensus accuracy of the minor contributor can fluctuate more due to the EM algorithm’s poor performance with imbalanced mixture ratios.

#### 3.3.4. Impact of ADO and ADI on Consensus Accuracies

**Figure 5:**
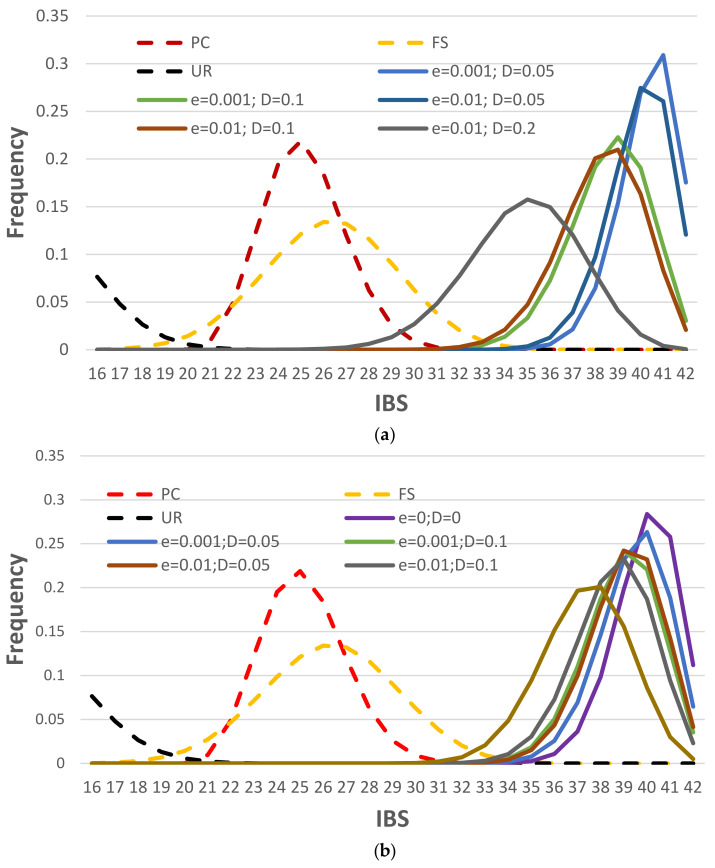
IBS distributions of unrelated and related pairs (UR, PC, and FS, in dotted lines) and pairs of the true profile versus the consensus profile with a given number of cells (in solid lines) for (**a**) 1 diploid cell and (**b**) 4 haploid cells. 1,000,000 simulations for each distribution. Missing alleles are excluded in counting IBS.

The above analysis used the typical ADO and ADI rates as an example (*D* = 0.2 and *e* = 0.01) to investigate the performance of consensus. The consensus accuracies are expected to be higher with lower ADO and ADI rates. Figure 5 shows the IBS distributions of the consensus profiles from one diploid cell (Figure 5a) and four haploid cells (Figure 5b), as examples, compared with the IBS distributions of UR, PC and FS. The IBS distribution of two diploid cell profiles was only slightly higher than that of one diploid cell profile, particularly when the ADO and ADI rates were low. Therefore, only one diploid cell profile scenario was used here to assess whether one diploid cell profile can provide high accuracies of determining the sources of contributors. If *D* = 0 and *e* = 0, the IBS should always be 42 for diploid cells. Thus, this scenario is not included in Figure 5a.

As expected, lower ADO and ADI rates provided higher IBS values on average. ADO had a larger impact on the distributions, mostly because the ADO rates were much larger than ADI rates in the simulated scenarios. Same as shown in Figure 3a, with *D* = 0.2 and *e* = 0.01, one diploid cell profile would be good enough to differentiate if it is from a known reference person or an unrelated person. If contributors are related, *D* ≤ 0.05 may be required to precisely differentiate a known individual from his/her full-siblings (i.e., ~0.15% average error rate with a threshold of IBS ≤ 34), although *D* = 0.1 may also be acceptable (i.e., ~0.8% average error rate with a threshold of IBS ≤ 32). With more cells sampled and clustered, the consensus accuracies are expected to be higher, which would tolerate higher ADO and ADI rates to obtain similar accuracies.

With four haploid cells, even without ADO and ADI, the full profile may not be able to be recovered in most cases. However, the consensus profile, even partial, can be differentiated from an unrelated person, even with an ADO rate of 0.2. To differentiate a known individual from his/her full-siblings, with four haploid cells in a single cluster, it would be desirable to have an ADO ≤ 0.05 (i.e., ~0.35% average error rate with a threshold of IBS ≤ 34).

**Figure 6:**
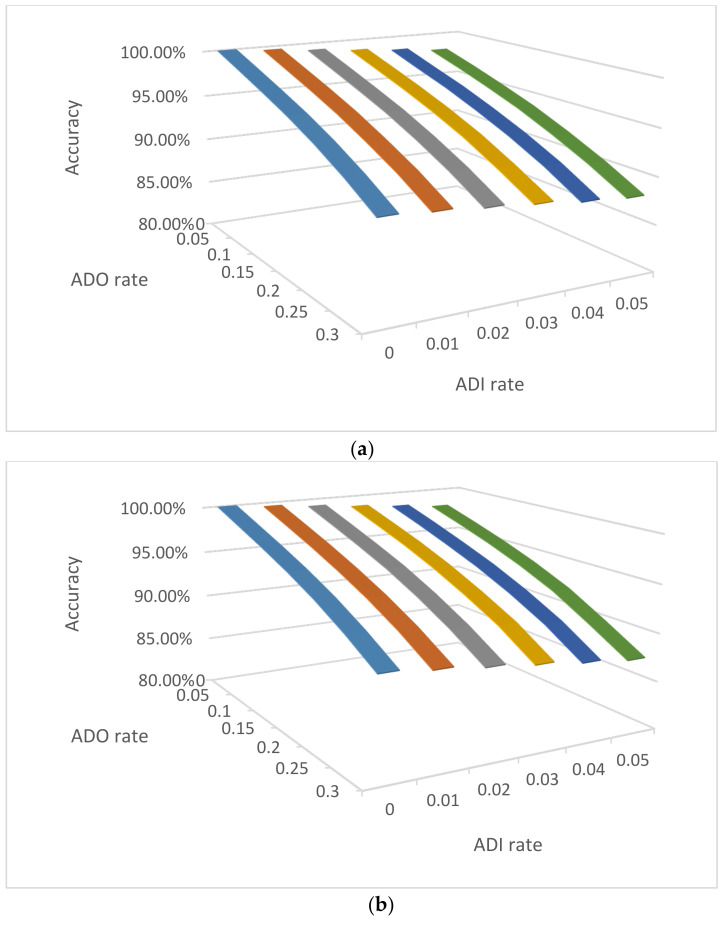

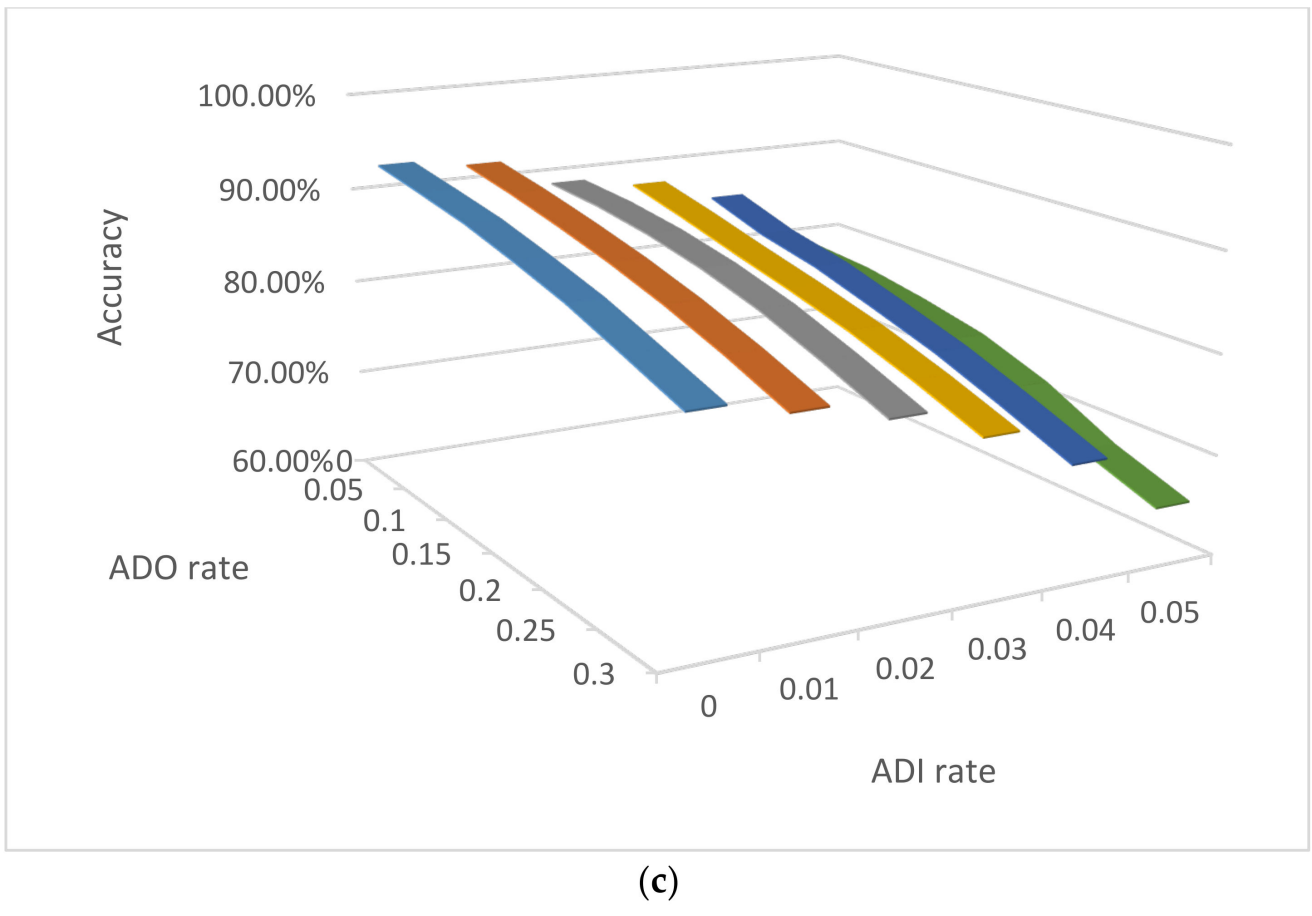
The accuracies of consensus for 3-person mixtures with 2, 6, and 12 single cells with various ADO and ADI rates in three scenarios: (**a**) diploid cells with three unrelated contributors, (**b**) diploid cells with three family trio contributors (father, mother, and child), and (**c**) haploid cells with three unrelated contributors.

Additionally, the same examples as in Figure 1 were simulated to assess the consensus accuracies with potential incorrectly clustered cells even with correct NOC estimations (Figure 6). The accuracies decrease with either increasing ADO or ADI rates. As expected, the accuracies of diploid cell mixtures decrease slower than those of haploid cell mixtures, because haploid cells have half the alleles of diploid cells. The ADI seems to have a bigger impact on haploid mixtures compared with diploid mixtures. Therefore, for genotyping mixture samples comprised of sperm, it would be better to have a low ADI rate, while a relatively high ADO rate may be acceptable.

In addition, similar to the data in Table 3, the unrelated and family trio diploid mixtures had almost identical consensus accuracies for diploid cells. In other words, although clustering may provide better NOC estimations for mixtures with unrelated contributors than family trio mixtures (i.e., 89.80% vs. 72.16%, respectively), if the NOC is correctly estimated, clustering performs similarly for the unrelated and family trio contributor mixtures. 

## 4. Discussion

### 4.1. A Paradigm Shift of Mixture Interpretation

With the emergence of single-cell technologies, a number of studies have employed these technologies for forensic applications, mostly on the wet-lab work to generate genotype profiles from single cells. The simulation studies herein are the first one to provide guidance on a comprehensive interpretation workflow for single-cell mixture profiles, evaluate the capabilities of single-cell profiles for mixture interpretation, and support that the single-cell based mixture interpretation can provide a precision that cannot be achieved by manual or the probabilistic genotyping interpretation approaches with DNA profiles generated by standard CE-STR analyses.

**Figure 7:**
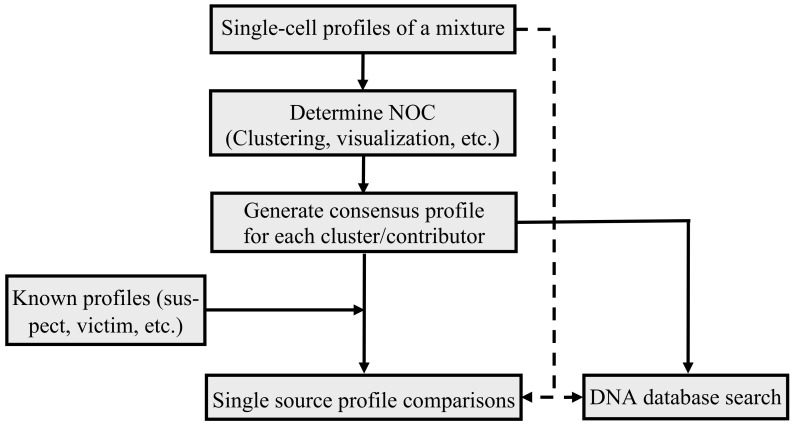
The interpretation workflow of single-cell profiles generated from a mixture.

Figure 7 illustrates the general mixture interpretation workflow with the single-cell mixture profiles. First, with the single-cell profiles generated from a mixture, the NOC of the single-cell profiles is estimated, either by clustering (e.g., EM algorithm and Silhouettes method), distance measure (e.g., IBS), or visualization. Second, the consensus profile of each cluster/contributor is generated from the single-cell profiles in each cluster. Then, the consensus profile(s) would be either directly searched against a DNA database(s) for partial or full matches (or hits), or compared with known profiles (e.g., suspect’s profile) to determine if these profiles, or these profiles’ relatives, potentially could be the contributor(s) of the mixture. In cases in which a full, or close to full profile, is obtained from a single cell(s), the profile may be directly compared with a known reference profile(s) or searched in a DNA database(s).

With single-cell technologies, a DNA mixture is not formed during the DNA extraction process. Thus, the interpretation is simplified and based on single source profile comparisons. Therefore, the need for methods, such as probabilistic genotyping, that indirectly determine the genotypes of the contributors, may not be required in some cases. Instead, the single-cell based approach can directly determine the profiles of the contributors from single-cell profile clusters or from the single source profiles of the individual cells.

For the single source comparisons to confirm or exclude individuals, IBS counting can be employed to determine the potential source or relationship between a consensus profile from single cells and a reference profile to be compared by a predefined IBS threshold as shown in Figure 3 and Figure 4. A LR based approach still can apply in these comparisons [58]. Let A be the consensus profile and B be a reference profile (e.g., a suspect’s profile). The hypotheses can be framed, for example, as A and B are from the same source or from different and unrelated sources. Then, the following equation can be used to calculate an LR, with ADO and ADI incorporated to explain the allele difference between A and B, if there are any. However, the ADO and ADI rates of the consensus profile are different from the rates for single cell profiles. These rates should be calculated by consensus similar to the methods of estimating quality scores in MPS [59].

(1) $$\mathrm{LR}\;=\frac{\mathrm{Pr}(A,B|A\;\&\;B\;\mathrm{are}\;\mathrm{from}\;\mathrm{same}\;\mathrm{source})}{\mathrm{Pr}(A,B|A\;\&\;B\;\mathrm{are}\;\mathrm{unrelated})}.$$

In Equation (1), the numerator is the product of the transition probabilities in Table 1 of [60] and the genotype frequency of B, and the denominator is simply the product of the genotype frequencies of A and B. When all alleles of A and B are identical and there is no ADO and ADI, this equation reduces to the inverse of the traditional Random Match Probability (RMP) of A or B.

In cases when a relative of, for example, the suspect is hypothesized as the contributor, both IBS and LR approaches can still apply. For the IBS approach, a different threshold can be used, as shown in Figure 3 and Figure 4. For the LR approach, the denominator hypothesis would be different (Equation (2)). The numerator can be calculated by expanding Table 1 in [61] with the incorporation of ADO and ADI, using a similar model to that described in [60].

(2) $$\mathrm{LR}\;=\frac{\mathrm{Pr}(A,B|A\;\&\;B\;\mathrm{are}\;\mathrm{related}\;\mathrm{as}\;a\;\mathrm{specific}\;\mathrm{relationship})}{\mathrm{Pr}(A,B|A\;\&\;B\;\mathrm{are}\;\mathrm{unrelated})}.$$

If there is no known profile to compare, the consensus profile (full or partial) may be searched against a forensic DNA database(s) (e.g., CODIS). If a consensus profile is generated from a cluster with a good number of sampled cells (e.g., ≥10), the partial matches (or hits) highly likely belong to family members of the sample donor or the sample donor.

### 4.2. The Capabilities and Limitations

This study supports that, based on the single-cell technologies, the DNA mixtures can be precisely deconvoluted with proper interpretation methods. With the single-cell profiles of any DNA mixture, the chance of missing a minor contributor can be quantified, and the proportion of each contributor in the mixture can be determined at the level of single cells or chromosomes through the single-cell profile clusters. These proportions may not reflect the true proportions in the whole mixture sample before cell isolation, particularly when the proportion is small or the number of sampled cells is small, because of cell extraction efficiency and sampling error, as only a small percentage of the cells may be isolated for genotype profiling. However, depending on the case scenario, mixture proportions may not be important or relevant compared with just identifying the cells that are present.

The probabilities of not detecting a minor contributor estimated in Table 2 assume a large number of cells are available for analysis. For low-quantity DNA samples, which may contain a few cells, hypergeometric distribution may be a better model to estimate these probabilities. However, the hypergeometric distribution requires knowing the total number of cells in a forensic sample, which may not be possible or precisely determined. In contrast, binomial distribution does not require the total number of cells in the estimation, and thus it may still be a good option in low-quantity DNA samples.

NOC estimation is the key component in this interpretation workflow. If the NOC is overestimated (e.g., a two-person mixture is determined as a three-person mixture), most likely the single cells of a contributor(s) are separated into two or more clusters, and the consensus profile of each cluster may still be valid. However, if the NOC is underestimated, cells from multiple contributors would be merged into one cluster, which could lead to an incorrect consensus profile. Nevertheless, by the clustering approach, the NOC of a mixture can be precisely determined for the mixtures with a high number of cells and relatively balanced mixture ratios, even for mixtures with related contributors.

In this study, for the tested classic clustering algorithms, EM performed better than K-Means in general, but K-Means performed better in some mixtures, such as the (5,5) mixture with unrelated contributors. Both of these classic algorithms do not perform well with imbalanced mixture ratios [54]. The EM algorithm does not explicitly require a distance measure, and the Euclidean distance was used in the K-Means algorithm. While the Euclidean distance measure may not be the best distance for this application, it was used herein as a proof of concept. Specifically designed distances for STR alleles, such as counting the number of step differences between STR alleles, only counting the heterozygous loci to mitigate the impact of ADO, or allele or genotype matching based measures (e.g., IBS), could be better choices for algorithms which require a distance measure. The Silhouettes method was used in this study to determine the number of clusters, but it does not work for the scenarios when NOC = 1 and is not necessarily the best method for single-cell STR data. Other methods, such as the Davies–Bouldin index, the Dunn index, and the Akaike information criterion (AIC), may help determine the number of clusters and may be tested in the future. Overall, better clustering algorithms (such as t-SNE clustering [62], semi-supervised and self-supervised learning [63,64]) and better methods of determining the number of clusters should be pursued for the single-cell profiles with imbalanced mixtures. In addition, similar to that of other single-cell applications, visualization can be helpful to estimate the NOC, as human judgment may do better deciding the NOC when the number of instances is small (e.g., few to hundreds), which is the case for many forensic mixture samples. However, human judgment can be subjective; different individuals may reach different conclusions. Thus, visualization is more of a tool to assist the users to decide NOC or limit the range of possible NOCs.

With a correct NOC, a high precision consensus profile can be obtained through the clustered cell profiles. The sources of these consensus profiles can be precisely determined by comparing them with the known profiles or considering the untyped relatives. The accuracy relies on the number of cell profiles in the clusters, as well as the ADO and ADI rates. But even with *D* = 0.2 and *e* = 0.01, the consensus accuracies of all contributors approach 100% for mixtures with ≥40 cells and relatively balanced mixture ratios. Particularly, the family trio mixtures can be deconvoluted to recover the genotypes of the individual contributors, even though related, which in theory is more challenging with CE-STR analysis without conditioning on one of the contributors [65]. This study did not investigate the consensus accuracies when the estimated NOC was incorrect. It was mainly because, if the number of the true profiles (e.g., two) and the number of consensus profiles (e.g., three) are different, it is not easy to decide which consensus profiles should compare with which true profiles. The accuracies with NOC overestimation and underestimation will be investigated in future studies.

The success of the single-cell mixture interpretations was based on the assumption that a reasonable number of intact single cells can be isolated from a DNA sample, there are no doublets or triplets of cells during cell isolation, and most of the alleles of these isolated single cells can be successfully genotyped or sequenced. Although these assumptions may not be held, low quality data per call could be filtered out using a threshold or with algorithms. For example, doublets can be detected by an outlier detection algorithms [66] or simply counting the number of alleles (i.e., triplet and/or quadruplet allele genotypes per locus) or heterozygotes. Regardless, the interpretation methods developed in this study allowed for imperfect data, such as small sample sizes, ADO, and ADI.

For some degraded forensic samples, many of the cells may be lysed, and thus isolating intact cells may be difficult. Additionally, if extracellular DNA is present, the DNA fragments released from the lysed cells may be isolated with an intact cell(s), which could cause extra alleles to appear for some of the single-cell profiles. Validation studies should be able to determine the impact of such events occurring. However, the impact may be small because the extracellular fragments are a small subset of the genome; forensic applications currently target only a very small percentage of the human genome (i.e., ~10k bp out of 3 billion bp). In addition, if the NOC is estimated correctly and the consensus works well, the drop-in alleles still may be able to be filtered out.

Even if a good number of cells are isolated, genotype profiling of the single copy alleles in each single cell can be challenging, depending on the ADO and ADI rates. The ADO and ADI rates can have a substantial impact on the analysis, such as NOC estimation and allele consensus. The current ADO and ADI rates observed in various studies [19,27,34] may still be high for each single-cell profile compared with the profiles generated from bulk cells. However, WGA could help enrich targets [31,33] for higher accuracies of genotype profiling, and single-cell isolation and amplification technologies are likely to become more robust.

Generating consensus profiles from multiple single cells that are clustered can mitigate the effects of high ADO and ADI rates, as described in Section 3.3. If there are ≥10 cell profiles in a cluster, with *D* = 0.2 and *e* = 0.01, a full consensus profile can be generated with no mismatched alleles or only one mismatched allele on average, with up to three mismatched alleles, in ~99.96% of the diploid mixtures and ~99.7% of the haploid mixtures. The process is also highly successful for developing the consensus profile among related profiles.

In addition, the NOC estimation and allele consensus with single-cell profiles do not need to assume independence among the tested alleles and markers, since the machine learning algorithms by nature are able to accommodate dependent variants. Therefore, in contrast to the complexity of current practices to incorporate dependence among the markers, the single-cell based mixture interpretation can directly use the genetically linked markers and markers in Linkage-Disequilibrium (LD) together, as well as the dependence among the alleles at the same locus, without any statistical corrections. This feature makes the single-cell based interpretation readily able to include as many markers as possible to increase the discriminating power and better identify individual contributors. However, current methods, including the current probabilistic genotyping approach, do not readily address dependent markers.

### 4.3. Future Improvements

As a proof-of-concept, this study only simulated the 21 STR loci in the GlobalFiler kit by simplified but reasonable ADO and ADI models to demonstrate the capabilities of using single-cell profiles to deconvolve mixtures. The same principles should apply for other autosomal markers as well, either more STRs, Single Nucleotide Polymorphisms (SNPs), or Insertion-Deletions (InDels). With more markers typed, the accuracy of NOC estimation should increase. ADO may have a higher impact on performance using SNPs than using STRs, since most of the SNPs have only two alleles at a locus and three possible genotypes at each locus. But the huge numbers of SNPs in the human genome should be able to overcome this limitation quite well. Indeed, whole genome sequencing (WGS) of single cells has been described for other applications [67].

The lineage markers on mitochondrial DNA (mtDNA) and the Y chromosome can be very useful for NOC estimation and allele consensus due to their unique features. The diploid cells from the same contributor should have the same Y chromosome markers (if a male contributor) and mtDNA haplotype (except for heteroplasmy or somatic mutations). Use of Y markers in the haploid cells is more complicated. Half of all sperm from a male contributor should have the same Y chromosome barring mutation, and the other half of the sperm should carry the same X chromosome barring mutation. Therefore, an interpretation process could be designed that first clusters profiles based on Y chromosome markers, followed by autosomal or X chromosome markers. X chromosome markers have been useful in certain cases, such as incest cases. However, mixture interpretation using X chromosome markers with CE-STR analysis has been problematic. With single-cell profiling, X chromosome markers could be used routinely for mixture deconvolution, especially when combined with Y markers.

The ADO model employed in this study did not consider degradation due to different allele and locus sizes. Accuracies could increase if degradation was included in the ADO model, such as including the pattern of the ADO rates for different sizes of alleles could improve the modeling of ADO rates for different alleles. A detection threshold still needs to be determined to differentiate alleles from noise. The ADI model used in this study assumed a random genotyping error while a large proportion of the ADIs are likely exaggerated stutters for specific STR alleles. It would be desirable to incorporate a stutter model, which should increase the accuracies of NOC estimation and allele consensus; less distinctly different alleles would be observed in each cluster with a stutter model allowing the profiles to cluster more effectively. However, not all wet-lab technologies would generate stutters. For SNPs and Indels, there is no stutter. For STRs, the stutters may be minimized by the recently developed Unique Molecular Index (UMI) technology [68]. There are many factors that may impact ADO and ADI, such as types of markers, sequencing platforms, library preparation kits, amplification kits, sequencing kits, protocols, software programs to call alleles, detection thresholds, and so forth. Thus, technology and marker specific ADO and ADI models may be the better options. In addition, the independence of the ADO and/or ADI events among the cells may not be assumed with real samples [69]. Therefore, more sophisticated ADO and ADI models that incorporate the underlying dependence between the cells may be better refracting the actual scenarios. Furthermore, this study used all single cells in analysis. In real casework, a filter should be implemented to remove the single cells with the number of missing alleles or loci beyond a certain threshold, and thus, only good quality data would be used in clustering and profile comparisons, which in general should improve interpretation accuracies.

Another improvement in accuracy could be to use clustering algorithms with a better performance on imbalanced data [70]. Particularly, when a minor contributor is represented with an extremely small number of sampled cells (e.g., one or two), outlier detection algorithms (e.g., Isolation Forest [71]) or self-supervised learning [72], instead of the classic clustering algorithms, may be used to select the single cell(s) of the minor contributor(s). Improving accuracies of determining the number of clusters, other NOC methods, such as Davies–Bouldin index, Dunn index, Akaike information criterion (AIC), and so forth, may also be considered. The current allele consensus method treats two alleles at the same locus independently. Performance may be improved if consensus is performed at the genotype level. With such improvements, it is reasonable to expect that this single-cell interpretation workflow should be able to interpret mixtures with a higher number of contributors (i.e., NOC ≥ 4).

In addition, the measures of several different NOCs may be very close to each other, and multiple alleles may be close likely being the consensus allele in the consensus profile. Picking one among multiple similarly likely NOCs or alleles could lead to error. Thus, the interpretation methods may allow multiple possible scenarios with estimated weights, similar to the continuous probabilistic genotyping approaches. These scenarios may be evaluated, compared, and combined in a Bayesian or LR fashion.

### 4.4. Costs

The whole mixture interpretation workflow with the current routine methods includes wet-lab work, data interpretation, judicial proceedings, law enforcement investigation, and so forth, and a cost benefit analysis should be considered in a systems fashion as opposed to solely the laboratory cost. The cost of the wet-lab workflow may be $50–100 per sample currently (including sample collection, DNA extraction, quantification, amplification, genotyping, but not including the labor), depending on the costs of the reagents and local infrastructure. There are more costs that tend to occur after the DNA mixture profile is generated, such as DNA interpretation (including software and hours of work interpreting mixture profiles), evidence presentation in the court, and so forth. Additional costs may also occur, including the law enforcement investigation, reanalyzing samples/aliquots, hiring external reviewers for complex mixture interpretation, and so forth.

Compared with the current routine methods, the single-cell based mixture interpretation methods will add more costs to the wet-lab workflow, depending on the number of cells to be analyzed and the reagents used in the workflow. In general, the more cells that are analyzed, the higher are the costs. However, the cost increase may not be linear to the number of cells to be tested, as the volume of reagents may be reduced in many steps (such as amplification), and some steps of the workflow will not be needed (such as extraction and quantification). More importantly, the single-cell based methods provide a much more simplified interpretation with higher precision. Thus, the costs of the subsequent work (such as data interpretation, additional wet-lab analysis, law enforcement investigation, external review, judicial proceedings, etc.) may be substantially reduced, and the overall costs of mixture cases, at a systems level, may be reduced.

Thus, while sampling multiple single cells will be more costly for the wet lab work than analyzing one mixture (i.e., pooled cell) sample, costs of analyses, such as MPS, continue to drop, and more facile single-cell technologies are likely to be developed. However, more importantly, the cost of analysis should be weighed against the value of reducing uncertainty in mixture interpretation, saving time and resource for law enforcement investigations and saving costs of judicial proceedings, which in turn would save the overall costs of an investigation by the government.

## 5. Conclusions

Overall, this study has demonstrated that the single-cell based mixture interpretation can provide a precision that cannot be achieved with current standard CE-STR analyses. A new paradigm for mixture interpretation is available to enhance the success of forensic genetic casework. More effort should be dedicated to improving the details of the interpretation methods, as well as the single-cell isolation and genotype profiling methods. In addition, as we see with other recent advances (such as familial searching or investigative genetic genealogy), this single-cell profiling approach could also be used after other approaches have not been able to achieve a desired outcome, assuming a sufficient sample for additional testing or by preserving part of the biological evidence to repeat the analyses with a different approach.

## Figures and Tables

**Table 1:** Accuracies of estimating the NOC with the EM algorithm and Silhouettes method for the various mixture scenarios. UR = unrelated, PC = parent-child, and FS = full-sibling. *D* = 0.2; *e* = 0.01; 10,000 simulations for each mixture scenario.

No. of Cells	2-Person Mixture							3-Person Mixture			
	Mixture	Diploid			Haploid			Mixture	Diploid		Haploid
		UR	PC	FS	UR	PC	FS		UR	Trio	UR
160	80,80	100.00%	100.00%	99.90%	99.90%	98.04%	92.16%	53,53,53	99.96%	88.32%	96.55%
	106,54	100.00%	99.98%	99.74%	99.88%	96.53%	89.27%	32,32,96	99.69%	85.64%	81.79%
	128,32	100.00%	99.85%	98.87%	99.16%	91.35%	81.70%	16,48,96	98.60%	78.46%	52.55%
	144,16	99.97%	98.54%	94.86%	95.97%	87.64%	81.28%				
	152,8	99.55%	91.59%	86.12%	92.43%	87.98%	83.97%				
80	40,40	100.00%	99.93%	99.91%	99.93%	98.73%	96.32%	26,26,26	99.97%	87.94%	96.44%
	53,27	100.00%	99.94%	99.78%	99.87%	98.17%	94.40%	16,16,48	99.68%	86.51%	82.47%
	64,16	99.99%	99.96%	99.53%	99.71%	94.37%	87.68%	8,24,48	98.36%	80.54%	54.15%
	72,8	99.96%	98.99%	96.55%	95.56%	85.95%	81.09%				
	76,4	99.67%	93.47%	88.56%	88.91%	83.54%	79.10%				
40	20,20	100.00%	99.99%	99.93%	99.72%	95.25%	92.41%	13,13,13	99.96%	88.29%	91.81%
	26,14	100.00%	99.93%	99.87%	99.76%	94.56%	90.23%	8,8,24	99.79%	86.06%	76.22%
	32,8	100.00%	99.89%	99.22%	99.18%	89.52%	81.95%	4,12,24	98.44%	79.79%	46.55%
	36,4	99.90%	98.03%	95.37%	93.40%	76.58%	71.13%				
	38,2	95.49%	78.98%	74.69%	84.31%	68.47%	65.89%				
20	10,10	100.00%	99.54%	99.02%	97.21%	82.30%	79.50%	6,6,6	99.81%	85.59%	69.19%
	13,7	99.98%	99.48%	98.84%	97.30%	82.77%	79.09%	4,4,12	99.52%	82.22%	51.34%
	16,4	99.96%	99.62%	98.92%	95.34%	73.85%	70.30%	2,6,12	89.80%	72.16%	21.69%
	18,2	99.61%	97.00%	94.29%	81.92%	59.54%	58.65%				
	19,1	98.51%	86.74%	82.46%	60.82%	53.45%	53.40%				
10	5,5	99.95%	96.77%	94.02%	82.78%	56.59%	55.61%	3,3,3	97.16%	66.81%	33.58%
	6,4	99.84%	96.65%	93.90%	80.38%	55.79%	53.41%	2,2,6	99.05%	76.26%	38.63%
	8,2	99.68%	94.61%	91.60%	78.59%	49.60%	48.24%	1,3,6	59.31%	37.65%	12.44%
	9,1	98.91%	89.19%	85.09%	65.71%	41.74%	42.05%				

**Table 2:** The probability of not detecting a minor contributor in a set of single-cell samplings.

No. of Cells	DNA Quantity (pg)		The Mixture Proportion of a Contributor				
	Diploid	Haploid	1%	5%	10%	20%	50%
1	6.6	3.3	99.00%	95.00%	90.00%	80.00%	50.00%
5	33	16.5	95.10%	77.38%	59.05%	32.77%	3.13%
10	66	33	90.44%	59.87%	34.87%	10.74%	0.10%
15	99	49.5	86.01%	46.33%	20.59%	3.52%	0.00%
20	132	66	81.79%	35.85%	12.16%	1.15%	0.00%
40	264	132	66.90%	12.85%	1.48%	0.01%	0.00%
80	528	264	44.75%	1.65%	0.02%	0.00%	0.00%
160	1056	528	20.03%	0.03%	0.00%	0.00%	0.00%
500	3300	1650	0.66%	0.00%	0.00%	0.00%	0.00%

**Table 3:** The average of the average accuracies of consensus alleles compared with the true alleles of the contributors. Only the mixtures with the correct NOC estimation were included. The NOC was determined using the EM algorithm and Silhouettes method. *D* = 0.2; *e* = 0.01; 10,000 simulations for each mixture scenario.

No. of Cells.	2-Person Mixture							3-Person Mixture			
	Mixture	Diploid			Haploid			Mixture	Diploid		Haploid
		UR	PC	FS	UR	PC	FS		UR	Trio	UR
160	80,80	100.00%	100.00%	100.00%	100.00%	99.93%	99.28%	53,53,53	100.00%	100.00%	100.00%
	106,54	100.00%	100.00%	99.99%	100.00%	99.80%	98.83%	32,32,96	100.00%	100.00%	99.94%
	128,32	100.00%	99.98%	99.90%	99.55%	98.64%	97.17%	16,48,96	99.96%	99.95%	99.47%
	144,16	99.85%	98.91%	97.91%	94.96%	93.92%	91.86%				
	152,8	98.01%	90.16%	89.05%	81.77%	85.70%	84.88%				
80	40,40	100.00%	100.00%	100.00%	100.00%	99.96%	99.73%	26,26,26	99.99%	99.99%	99.97%
	53,27	100.00%	100.00%	99.99%	99.99%	99.90%	99.49%	16,16,48	99.91%	99.91%	99.73%
	64,16	99.94%	99.93%	99.89%	99.75%	99.09%	97.90%	8,24,48	99.47%	99.45%	98.53%
	72,8	99.19%	98.65%	98.01%	95.15%	92.18%	90.78%				
	76,4	96.40%	93.09%	91.63%	81.77%	83.45%	83.02%				
40	20,20	99.96%	99.96%	99.96%	99.93%	99.80%	99.52%	13,13,13	99.69%	99.68%	99.00%
	26,14	99.88%	99.87%	99.87%	99.67%	99.41%	98.90%	8,8,24	98.94%	98.95%	97.56%
	32,8	99.21%	99.18%	99.08%	98.37%	96.61%	95.43%	4,12,24	97.79%	97.78%	94.55%
	36,4	96.79%	96.17%	95.85%	91.16%	87.64%	86.57%				
	38,2	85.79%	81.23%	81.22%	76.48%	78.91%	78.88%				
20	10,10	99.16%	99.17%	99.13%	98.20%	96.75%	95.68%	6,6,6	96.98%	96.97%	92.68%
	13,7	98.90%	98.87%	98.85%	97.53%	95.57%	94.51%	4,4,12	95.72%	95.71%	89.05%
	16,4	96.83%	96.80%	96.71%	93.02%	89.51%	88.51%	2,6,12	94.02%	94.02%	84.83%
	18,2	92.78%	92.31%	92.01%	81.99%	77.81%	78.35%				
	19,1	90.85%	88.80%	87.86%	69.30%	68.57%	69.78%				
10	5,5	96.33%	96.34%	96.30%	91.56%	87.57%	86.61%	3,3,3	92.68%	92.74%	78.80%
	6,4	95.37%	95.33%	95.33%	90.70%	86.72%	85.95%	2,2,6	89.45%	89.47%	75.58%
	8,2	92.11%	91.99%	91.97%	82.58%	78.46%	77.78%	1,3,6	90.86%	90.42%	66.12%
	9,1	90.81%	90.36%	90.17%	71.50%	67.26%	67.31%				

## Data Availability

No data to report.

## Supplementary Materials

The following are available online at www.mdpi.com/article/10.3390/genes12111649/s1, Supplementary Table S1. A simulated mixture example with two unrelated contributors (20 diploid cells each) in the ARFF format defined by WEKA. “?” = missing or drop-out allele. Supplementary Table S2. Accuracies of NOC with K-Means algorithm and Silhouettes method for various mixture scenarios. UR = unrelated, PC = parent-child, and FS = full-sibling. Supplementary Figure S1, which includes the rotatable plots of Figure 2.
