# Supplementary material for: Precision DNA Mixture Interpretation with Single-Cell Profiling
Source: Genes (Basel). 2021 Oct 20;12(11):1649. doi: 10.3390/genes12111649 (PMC8623868; doi:10.3390/genes12111649)
Supplement: Supplementary file 1 [file genes-12-01649-s001.zip › Supplementary Figure S1.c.html]

Figure S1.c (2,6,12) 3-person haploid cell mixture
